# Fatal Lactic Acidosis in a Kidney Transplant Recipient on Combination Antiretroviral Therapy after Initiation of Tacrolimus Therapy

**DOI:** 10.1155/2011/210178

**Published:** 2012-01-05

**Authors:** Michael V. Holmes, Ranjababu Kulasegaram, Sebastian B. Lucas, Terry Wong, Rachel Hilton

**Affiliations:** ^1^Directorate of Nephrology, Transplantation and Urology, Guy's Hospital, Guy's and St Thomas' NHS Foundation Trust, London SE1 9RT, UK; ^2^Department of HIV and Genitourinary Medicine, Guy's and St Thomas' NHS Foundation Trust, London SE19RT, UK; ^3^Department of Histopathology, Guy's and St Thomas' NHS Foundation Trust, London SE19RT, UK; ^4^Department of Gastroenterology, Guy's and St Thomas' NHS Foundation Trust, London SE19RT, UK

## Abstract

In general, kidney transplantation is safe and efficacious in patients receiving treatment for HIV. Although multiple drug interactions between antiviral and immunosuppressive treatments exist, few patients experience serious adverse reactions. We report a case of fatal lactic acidosis in a healthy kidney transplant recipient with stable HIV infection who had previously received treatment for and cleared hepatitis C virus infection. Death occurred less than one month following the initiation of tacrolimus therapy. Based on predicted drug interactions, appropriate tacrolimus dosing was calculated prior to its commencement, yet plasma tacrolimus levels were initially unexpectedly high. The patient subsequently developed lactic acidosis and hepatic steatosis, presumably due to mitochondrial toxicity from the antiretroviral regimen on which he had previously been stable. We suspect CYP2C19*2 (poor metaboliser) genotype status and concomitant treatment with lansoprazole, tacrolimus, and antiretroviral (ARV) medications resulted in hepatic decompensation. This highlights the importance of careful interaction screening for all new drugs administered to patients with HIV who have complex treatment regimens as well as heightened clinical vigilance for unexpected toxicities.

## 1. Introduction

Since the introduction of highly active antiretroviral therapy (HAART) in 1996, mortality in patients with human immunodeficiency virus (HIV) infection has decreased markedly [1]. As a result, morbidity from other chronic conditions such as kidney, liver, and heart disease is increasing. Patients with HIV are at particular risk for development of chronic kidney disease, most notably HIV-associated nephropathy (HIVAN), which is the third most common cause of end-stage kidney disease in black individuals aged 20–64 years in the United States [2]. The presence of HIV was historically regarded as a contraindication to transplantation [3] because of the concern regarding the potential worsening of HIV disease and the increased risk of opportunistic infection by immunosuppression. However, current data suggest that transplant recipients with optimal control of HIV do as well in the short term as those without HIV, provided there is proper donor selection and recipient management [4–6]. Indeed, survival for patients with end-stage kidney disease and HIV is better after transplantation than on maintenance haemodialysis [7].

Concerns remain, however, regarding the concomitant use of antiretroviral medications and immunosuppressive agents that may be substrates for, or may induce or inhibit pharmacokinetic enzymes involved in drug handling, such as P-glycoprotein flux transporters or cytochrome P450 metabolizing enzymes found in the gut and liver. These interactions can lead to unexpected increases or decreases in drug plasma levels leading to drug toxicity, transplant rejection, or HIV disease breakthrough. Significant drug interactions have been observed depending on the class of antiretroviral agent [8]. Clearly, predicting such interactions is mandatory to inform decisions regarding drug dosing and administration. However, because as yet relatively few patients with HIV have received organ transplants, there remains scope for emergence of presently unrecognised drug interactions due, for example, to rare genotypes encoding pharmacokinetic enzymes of high or low activity and/or idiosyncratic reactions. We report here a kidney transplant recipient who was HIV positive with cleared hepatitis C (HCV) from previous treatment who developed unpredicted mitochondrial toxicity leading to hepatic microvesicular steatosis and fatal lactic acidosis following initiation of tacrolimus immunosuppression.

## 2. Case Presentation

A 39-year-old Caucasian male with chronic kidney disease due to a combination of kidney stones and high blood pressure was found to have HIV infection and commenced HAART in 1995 consisting of abacavir, nevirapine, and lamivudine, on which he achieved reconstitution of CD4 count (500 cells/*μ*L) and undetectable HIV RNA viral load (<40 copies/mL). 11 years later, he developed end-stage kidney disease and started peritoneal dialysis. Later that year, he was diagnosed with acute HCV genotype 1 infection and was treated with sustained virological response. Liver function tests at baseline were normal (Table 1). Neither liver biopsy nor transient elastography was performed. Other comorbidities included hypercholesterolaemia treated initially with bezafibrate and subsequently atorvastatin.

Eighteen months after starting dialysis, he was referred to this hospital and received a living related donor kidney transplant with a one-HLA haplotype match between donor and recipient. Immunosuppression consisted of basiliximab induction, ciclosporin microemulsion, mycophenolate mofetil, and oral prednisolone.

Six months after engraftment, there was a rise in serum creatinine above baseline, and he underwent a biopsy of his transplanted kidney which showed acute transplant rejection. This was treated with three consecutive daily doses of intravenous methylprednisolone without immediate improvement in kidney function. A decision was therefore made to switch the base immunosuppressive therapy from ciclosporin to tacrolimus. Tacrolimus is primarily metabolized in the liver by cytochrome P450 (CYP3A), and nevirapine is a slight inducer of CYP3A [9], thus by co-administering tacrolimus and nevirapine, it was predicted that plasma levels of tacrolimus would be decreased. The patient commenced tacrolimus at a standard dose (0.1 mg/kg twice daily). The initial serum tacrolimus level after 48 hours was very high (60 *μ*g/L), and the patient complained of tremor and weakness consistent with tacrolimus toxicity. Dosing was adjusted accordingly.

Two weeks later, the patient required hospital admission with malaise, metabolic acidosis, and deranged kidney and liver function (Table 1). Bacterial septicaemia was excluded by duplicate sets of blood and urine cultures, which were negative. HCV RNA was not detected. Abdominal examination, liver ultrasonography, and abdominal CT were unremarkable. Over the course of the next 12 hours, he became drowsy, hypotensive, and peripherally vasoconstricted despite fluid resuscitation. Arterial blood gas analysis revealed severe metabolic acidosis with a serum lactate level beyond the recordable range of the blood gas analyser (>20 mmol/L). HAART and immunosuppressive agents were discontinued at this stage. He required escalating doses of inotropic and vasopressor support and in spite of 48 hours of high-volume (65 mL/kg) continuous venovenous haemofiltration (CVVHF), he remained acidotic with high serum lactate levels (16 to >20 mmol/L). He suffered a cardio-respiratory arrest from which he was resuscitated. Sedation was withdrawn to assess his neurological function, but no cortical function and limited brain stem reflexes were elicited consistent with severe brain injury. Given his poor cerebral function and clinical prognosis, active treatment was withdrawn, and he died shortly afterwards.

**Figure 1:**
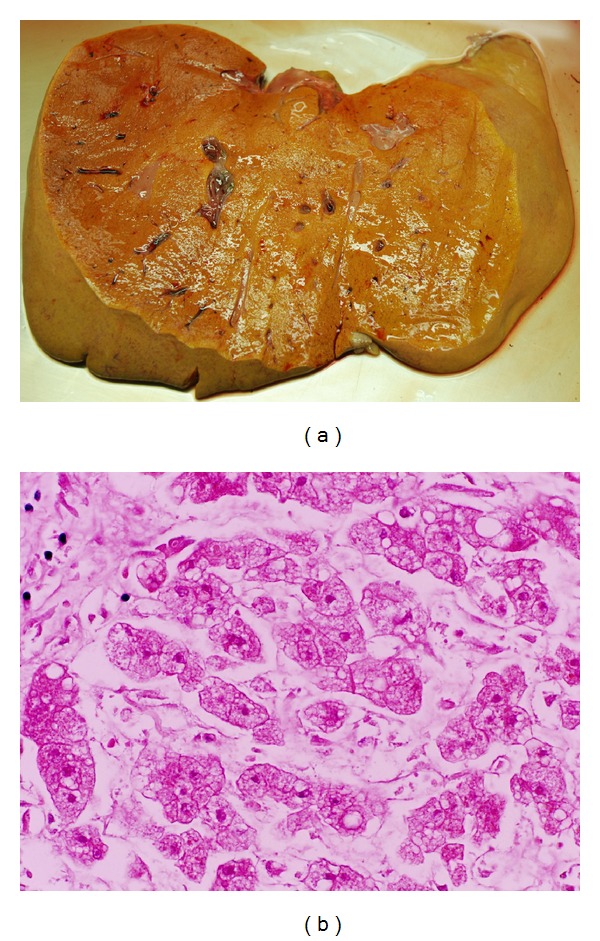
(a) The liver showing yellow steatosis. (b) Microvesicular steatosis in hepatocytes (H&E ×400).

At autopsy, the main pathology was in the liver. Weighing 1670 gm, it was soft, bright yellow, not fibrotic, and without focal lesions (Figure 1(a)). Histopathologically, there was global microvesicular steatosis (Figure 1(b)), no hepatitis, and minimal fibrosis (Ishak stage 1-2/6; some portal fibrosis and focal spurs of connective tissue in the lobules but absence of portal-portal bridging or diffuse perisinusoidal fibrosis). The other organs showed nonspecific changes of multi-organ failure. The steatosis was typical for toxicity associated with NRTI drugs.

 Antiretroviral drug levels tested posthumously on stored serum samples lay within the therapeutic range (Table 1). Genotyping demonstrated homozygosity for variants in exon 5 of the CYP2C19 gene (CYP2C19*2) rendering this enzyme nonfunctional.

## 3. Discussion

### 3.1. Drug Interactions between Immunosuppressive Drugs and Antiretroviral Medication

Interactions between immunosuppressive therapy and antiretroviral medications (ARV) are common [10]. The most notable occurs as a consequence of induction or inhibition of cytochrome P450 metabolizing enzymes and P-glycoprotein flux transporters found in the liver and intestine. The CYP3A subfamily is the most highly expressed of the cytochrome P450 enzymes, and CYP3A4 is the most abundant isoform. CYP3A4 is responsible for the metabolism of calcineurin inhibitors (CNI; cyclosporin and tacrolimus), mammalian target of rapamycin (mTOR) inhibitors (sirolimus and everolimus), and HIV protease inhibitors (PI). CNIs and PIs are not only substrates but also inhibitors of CYP3A4 and tend to increase systemic blood levels of CYP3A4 substrates, whereas some nonnucleoside analogue reverse transcriptase inhibitors (NNRTIs) such as efavirenz induce CYP3A, so increasing metabolism and decreasing blood levels of CYP3A4 substrates. In addition, many of these drugs are also substrates and inhibitors of P-glycoprotein (ATP-binding cassette, PGP), a membrane-bound efflux protein involved in drug transportation across cell membranes. In the intestine, PGP limits oral bioavailability of drugs by expelling them from the interior of enterocytes into the gut lumen. Thus, coadministration of inhibitors and substrates of both P-glycoprotein and CYP3A4, such as CNIs with PIs, would be expected to increase both CNI and PI uptake and systemic blood levels. Although the extent of these drug interactions is not fully characterized for all available therapeutic combinations, careful anticipation of appropriate drug dosing is essential prior to organ transplantation to minimize the risk of treatment failure or drug toxicity. In the case reported here, the patient was not receiving a PI. The only significant predicted drug interaction was an anticipated decrease in tacrolimus plasma concentration due to coadministration of nevirapine. 

### 3.2. Tacrolimus and Proton Pump Inhibition

Proton pump inhibitors such as lansoprazole are metabolized by both CYP2C19 and CYP3A4 isoforms of cytochrome P450. This leads to potential drug interactions with other substrates of CYP3A4 (such as tacrolimus), particularly when CYP2C19 activity is impaired [11]. Possession of the G→A allelic variation at the single nucleotide polymorphism rs4244285 is a marker of CYP2C19*2 status. This mutation alters the mRNA reading frame, resulting in a premature stop codon, yielding a truncated nonfunctioning protein (and consequent low phenotypic CYP2C19 enzymatic activity). In Caucasians, homozygosity for the risk allele (A/A) is rare (5%). Posthumous genotyping revealed that our patient was homozygous for CYP2C19*2, rendering this enzyme nonfunctional. This resulted in competition between lansoprazole and tacrolimus for CYP3A4-mediated metabolism and accounted for the unexpectedly high tacrolimus levels.

### 3.3. Lactic Acidosis and ARV

Lactic acidosis is a rare but potentially life-threatening condition associated with exposure to nucleoside/tide reverse transcriptase inhibitors (NRTIs), particularly stavudine and didanosine, although reported with all NRTIs. Other reported risk factors include female gender, pregnancy, obesity, prolonged ARV duration, and use of ribavirin with didanosine in patients with hepatitis C coinfection [12]. The cause is inhibition of human mitochondrial polymerase-*γ* resulting in impaired synthesis of mitochondrial enzymes that generate adenosine triphosphate (ATP) by oxidative phosphorylation. Although 20–60% of patients on NRTI therapy have elevated lactate levels, only 0.1% to 0.4% develop lactic acidosis [13], which may be difficult to diagnose because the presenting symptoms (e.g., lethargy, nausea, vomiting, and abdominal pain) may be nonspecific. The liver is consistently involved, and pathological examination usually reveals steatosis, which may be macrovesicular, microvesicular, or mixed [14]. Treatment consists of prompt recognition and discontinuation of the offending NRTIs. Several adjunctive therapies have been tried, with limited success, including essential vitamin coenzymes (thiamine and riboflavin), ubiquinone, biotin, zinc picolinate, *n*-acetylcysteine, uridine, and l-carnitine [15]. The mortality rate is almost 60% [16]. Other than prolonged use of ARV and the presence of HCV (discussed below), in the patient we report here, there were no obvious predictors of a heightened risk of lactic acidosis.

### 3.4. HCV and HIV Coinfection in Transplant Recipients

Due to similar routes of transmission, coinfection of HIV with other sexual and blood-borne viruses such as hepatitis B virus (HBV) and HCV is relatively common. Of the 35 million people living with HIV worldwide, around 20% have chronic HCV infection. The mechanism of interaction between the two viruses and their impact on liver injury is not completely understood, but patients with HIV are less likely to achieve spontaneous HCV clearance and progress more rapidly to liver fibrosis and end-stage liver disease than HIV-negative individuals. Furthermore, HIV/HCV coinfected patients are more susceptible to liver toxicity and steatosis from ARV, more so with the use of NRTIs, and in such patients, hepatic steatosis is associated with more advanced hepatic fibrosis [17]. Risk factors for hepatic steatosis in HIV-HCV coinfected patients include white race, increasing age, higher body mass index, insulin resistance, lower levels of high-density lipoprotein cholesterol, presence of lipodystrophy, HCV genotype 3, higher HCV plasma viral load, stavudine or didanosine use, and raised serum ferritin [18]. Among liver transplant recipients, poorer outcomes have been observed in patients coinfected with HIV and HCV in comparison with HIV or HCV monoinfected patients [6, 19], principally due to recurrent HCV infection. HCV-positive kidney transplant recipients have equivalent patient but decreased graft survival in comparison with HCV-negative recipients, poorer graft outcome being partly explained by mesangioproliferative glomerulonephritis in the allograft [20].

In the case we report here, although the patient had cleared HCV and remained HCV RNA negative with normal liver function tests, he had not had assessment of liver fibrosis by biopsy or transient elastography prior to kidney transplantation. Features which argue against a causal role of HCV in this case are sustained virological response to HCV eradication therapy; presence of Ishak stage 1-2 hepatic fibrosis on autopsy (a sign of early fibrosis often present in the general population); microvesicular steatosis, which is unrelated to HCV and consistent with a drug-induced lactic acidosis (typically NRTI); an AST-to-platelet ratio index (APRI) of 0.08, measured when the patient was stable, indicating haematological and biochemical absence of fibrosis.

### 3.5. In Search of Occam's Razor

The close temporal association between the initiation of tacrolimus and the onset of progressive liver dysfunction suggests a causal relationship, but the mechanism for this remains unexplained. The unexpectedly high tacrolimus levels probably resulted from CYP2C19*2 status and concomitant lansoprazole, both of which affected tacrolimus metabolism by CYP3A4. However, this does not explain the mitochondrial uncoupling with lactic acidosis and steatosis that occurred shortly after tacrolimus initiation; there are no previous reports of tacrolimus-induced lactic acidosis. It is possible that a steatosis diathesis was created by existing treatment with NRTI, which, coupled with the presence of elevated tacrolimus levels, resulted in hepatic decompensation. Yet, serum levels of HAART were normal (although this may not accurately reflect intrahepatic levels, nor is it indicative of increased risk of mitochondrial oxidative uncoupling). An alternative hypothesis is that the unique combination of genetic variation and polypharmacy precipitated hepatic decompensation through unknown pathways.

Organ transplantation for patients with HIV remains a novel therapy [21] and carries a risk of unanticipated drug interactions that may cause increased drug toxicity or diminished therapeutic effect. While these may shed new light on mechanisms of drug metabolism and disposition, our case highlights the need for careful interaction screening of all new treatment regimens in such patients as well as heightened clinical vigilance for unexpected toxicities.

## Figures and Tables

**Table 1:** Haematology, biochemistry, thrombosis, and virology test results at various time points prior to and following renal transplantation.

Variable	Reference range (min, max), adults	Pretransplant	4–6 weeks prior to tacrolimus initiation	One week following tacrolimus initiation	Two weeks following tacrolimus initiation
*Haematology*					
Haemoglobin (g/dL)	13.0, 17.0	11.8	12.5	12.5	12.9
White cell count (×10^9^)	4.0, 11.0	9.0	8.8	12.6	10.9
Platelet count (×10^9^)	150, 400	268	308	255	229
CD4 Lymphocytes (per *μ*L)	300, 1400	1188	956	235	21

*Coagulation*					
Activated partial thromboplastin time ratio	0.85, 1.16	0.91	1.08	1.23	1.51
International normalized ratio	0.90, 1.10	1.03	1.04	1.10	1.59

*Clinical Chemistry*					
Sodium (mmol/L)	135, 145	133	136	134	130
Potassium (mmol/L)	3.5, 5.0	4.3	4.3	4.6	5.1
Bicarbonate (mmol/L)	22, 30	25	26	19	15
Urea (mmol/L)	1.7, 8.3	22.0	7.8	14.4	14.1
Creatinine (*μ*mol/L)	59, 104	1415	156	206	233
Albumin (g/L)	40, 52	46	46	37	32
Alkaline phosphatase (IU/L)	35, 129	62	205	236	383
Bilirubin (*μ*mol/L)	0, 16	7	6	10	44
Alanine transaminase (IU/L)	4, 59	36	20	65	141
Gamma-glutamyl transferase (IU/L)	4, 72	42	61	354	1500
Creatine kinase (IU/L)	0, 229	Not measured	Not measured	Not measured	1468
Lactic acid (mmol/L)	0.4, 2.2	Not measured	Not measured	Not measured	18

*Virology*					
Hepatitis C RNA (IU/mL)		< 15	Not measured	Not measured	<15
HIV-1 RNA (copies/mL)		<40	<40	Not measured	<40
CMV DNA (copies/mL)		Not applicable	Not detected	Not detected	Not detected

*Drug levels*					
Ciclosporin level (*μ*g/L)	100, 200	Not applicable	74	Not applicable	Not applicable
Tacrolimus level (*μ*g/L)	8, 12	Not applicable	Not applicable	26	13

*Antiviral medications*		6 weeks prior to transplantation	15 weeks following transplant: on ciclosporin	NR	2 weeks after switching to tacrolimus
Abacavir (mean steady state ^§^C_min_, C_max_; ng/mL)	10, 3000	1551	1611	NR	538
Lamivudine (mean steady state ^†^C_min_, C_max_; ng/mL)	90, 1200	794	1615	NR	717
Nevirapine (therapeutic range min, max; ng/mL)	3000, 8000^‡^	5227	Not measured	NR	5488

Estimated from therapeutic doses of  ^§^300 mg abacavir twice daily and ^†^150 mg lamivudine twice daily (values derived from Delphic Laboratories (Liverpool) ltd.).
